# Transcatheter Patent Foramen Ovale Closure Is Effective in Alleviating Migraine in a 5-Year Follow-Up

**DOI:** 10.3389/fneur.2019.01224

**Published:** 2019-11-19

**Authors:** Yao-De He, Xiu-Li Yan, Chen Qin, Peng Zhang, Zhen-Ni Guo, Yi Yang

**Affiliations:** ^1^Stroke Center, Department of Neurology, The First Hospital of Jilin University, Changchun, China; ^2^Clinical Trial and Research Center for Stroke, Department of Neurology, The First Hospital of Jilin University, Changchun, China

**Keywords:** migraine, right-to-left shunt, patent foramen ovale, patent foramen ovale closure, treatment

## Abstract

**Background:** The association between patent foramen ovale (PFO) and migraine has been reported. However, whether transcatheter PFO closure is effective in alleviating migraine remains controversial. The objective of this study was to investigate the efficacy of PFO closure in alleviating migraine in a 5-year follow-up.

**Methods:** Migraineurs with PFO from 2013 to 2015 were included and divided into PFO closure group and non-PFO closure group according to their therapy. Contrast-enhanced transcranial Doppler (c-TCD) was performed to evaluate the degree of the right-to-left shunt (RLS), and headache impact test (HIT-6) questionnaire was administered to assess the disability of migraine at 1- and 5-year follow-up.

**Results:** Of 192 patients, 91 patients underwent PFO closure, and 101 patients refused. The HIT-6 scores of patients in the PFO closure group were significantly lower than those of the non-PFO closure group at both 1- and 5-year follow-up. These results were more pronounced in patients younger than 45 years. Furthermore, in patients with large RLS, the HIT-6 scores of patients in the PFO closure group were significantly lower at both 1- and 5-year follow-up compared with those of the non-PFO closure group. However, in patients with moderate RLS, this difference was significant only at 5-year follow-up.

**Conclusions:** PFO closure is effective in alleviating migraine in the long term. This effect is more obvious when patients are younger than 45 years and RLS is large.

## Introduction

Migraine is a common disabling neurological disorder characterized by recurrent unilateral, pulsing headache often in association with photophobia, phonophobia, and nausea. Migraine affects 15.3% of adult population in the United States (1) and 9.3% of Chinese adult population (2), and it exerts a negative impact on patients' daily activities, thus adding a great socioeconomic burden to the society (3). Over the past several decades, migraine, especially migraine with aura, has been linked with patent foramen ovale (PFO) (4–8). Recently, a multicenter case control study has further confirmed this association between PFO and migraine both with and without aura in the Chinese population (9). The relationship between migraine and PFO is explained by the theory that vasoactive substances, which are normally removed in the pulmonary circulation, may bypass the lung filter in the presence of an abnormal right-to-left shunt (RLS) and enter the cerebral circulation, eventually resulting in a migraine attack (10).

Transcatheter PFO closure has been proposed to be a new target for treating migraine. Nevertheless, research studies on the effect of transcatheter PFO closure on migraine have not yielded consistent results. Multiple studies have found that the symptoms of migraine were improved after PFO closure indicated by other diseases (e.g., paradoxical embolism and decompression illness) (11–15). Paradoxically, the Migraine Intervention with STARFlex Technology (MIST), Percutaneous Closure of PFO in Migraine With Aura (PRIMA), and Prospective, Randomized Investigation to Evaluate Incidence of Headache Reduction in Subjects with Migraine and PFO Using the AMPLATZER PFO Occluder to Medical Management (PREMIUM) trials did not find a significant effect of PFO closure on their primary endpoints (16–18). In addition, with a range from 6 months (16) to 45 months (14), the follow-up time in different studies was variable. Hitherto, only one non-randomized study of transcatheter PFO closure for the treatment of migraine with a follow-up time of 1 year in the Chinese population has been published (19).

In this present study, we aimed to assess the efficacy of transcatheter PFO closure in treating migraine by comparing the severity of migraine at different timepoints during a 5-year follow-up between patients who received the surgery and controls. Subgroup analyses were also performed to further investigate the possible indications of transcatheter PFO closure for migraineurs from a long-term point of view.

## Materials and Methods

The Ethics Committee of the First Hospital of Jilin University approved the study design. Written informed consent was obtained from all participants.

### Participants

We retrospectively studied the information of all newly diagnosed migraineurs with RLS confirmed by contrast-enhanced transcranial Doppler (c-TCD) and PFO confirmed by echocardiography in the Department of Neurology, the First Hospital of Jilin University from January 2013 to January 2015. The diagnosis of migraine was made by a neurologist on the basis of the International Classification of Headache Disorders III-beta (20). All included migraineurs were refractory or unwilling to take regular analgesics for migraine (e.g., NSAIDS, opioid analgesics, or triptans) and none of them had reported medication overuse or consumed preventive medications for migraine. We made a comprehensive explanation of the potential benefits and risks of transcatheter PFO closure to the patients and according to their consent of this procedure, they were separated into the PFO closure group and the non-PFO closure group. Patients who had RLS under grade II (categorization system is described in the following section), central nervous system disorders other than migraine, or contraindications of transcatheter PFO closure were not included in this study. The details of methodology of transcatheter PFO closure has been described and the safety of this procedure has been proved in our previous work (19). The follow-up lasted for 5 years. Throughout the follow-up, patients in both groups were suggested to use preventive treatments if their symptoms were not alleviated, and the proportions of different preventive treatments used by the subjects were recorded.

### HIT-6 Score

We performed the headache impact test (HIT-6) questionnaire to assess the disability of migraine. The HIT-6 questionnaire is a six-item self-report to assess the negative influence of headache on patients' daily activities. The HIT-6 score ranges from 36 to 78; a higher HIT-6 score represents a more severe headache, while an HIT-6 score of 36 represents no headache at all. In both groups, HIT-6 scores were collected at baseline by the same neurologist who diagnosed migraine. During the follow-up, the HIT-6 questionnaire was completed by a neurologist who was blinded to the group information over telephone for each patient at 1 year (±1 month) and 5 years (±1 year) after baseline, respectively. Simultaneously, patients in the transcatheter PFO closure group were asked to reexamine c-TCD to assess residual shunts.

### c-TCD Examination

c-TCD examinations were performed by an experienced sonographer with a Multi-DopX4 TCD detector (DWL, Sipplingen, Germany). The left middle cerebral artery (MCA) was monitored with the patient in a supine position. The contrast agent was composed of 9 mL saline solution, 1 mL air, and a drop of the patient's blood (21). After back and forth mixing for 30 cycles between two 10-mL syringes through a three-way stopcock to produce microbubbles (MB), the contrast agent was injected as a bolus through an 18-gauge needle inserted into the cubital vein of the patient. Testing was performed once at rest and twice during the Valsalva maneuver (VM). An MB was defined as a visible and audible (click, chirp, or whistle), short-duration, high-intensity signal within the Doppler flow spectrum. On the basis of the existing categorization systems (9, 22, 23), we adopted a 5-grade scale according to MB appearance in the TCD spectrum using unilateral MCA monitoring: negative = no occurrence of MBs; grade I = 1–10 MBs; grade II = 11–25 MBs; grade III > 25 MBs, but no curtain; grade IV = curtain (single MBs were indistinguishable within the TCD spectrum). As described elsewhere (9), grade II was defined as moderate RLS and grade III and grade IV were combined and defined as large RLS. RLS was considered constant if MBs were detected at rest and as provoked if MBs were detected only after the VM.

### Statistical Analysis

Statistical Package for the Social Sciences Version 17.0 (SPSS, IMB, West Grove, PA, USA) was used to perform the statistical analysis. Continuous data that complied with normal distribution were expressed as mean and standard deviation and were compared using Student's *t*-tests. Continuous data that did not comply with normal distribution were presented as median and quartiles and Mann Whitney *U*-test was performed for comparison. Discrete variables were expressed as the rate (percentage) and were analyzed using chi-square test. Statistical significance was considered if the calculated two-tailed *p*-value was < 0.05.

## Results

A total of 192 migraineurs with confirmed moderate-to-large RLS caused by PFO were included. Of those, 91 patients consented and underwent transcatheter PFO closure, 101 patients refused to undergo transcatheter PFO closure and thus were categorized into the non-PFO closure group. The baseline demographic and clinical characteristics of all migraineurs are shown in Table 1. As indicated, all listed terms (age, gender, course of migraine, migraine with or without aura, the shunt degree and status of RLS, and baseline HIT-6 scores) between the two groups were similarly matched.

**Table 1:** Demographic and clinical characteristics of all migraineurs at baseline.

	**PFO closure group (*n* = 91)**	**non-PFO closure group (*n* = 101)**	**t/Z/ χ^2^**	***p***
Age, years	37.1 ± 12.8	39.2 ± 12.1	1.180	0.239
Females patients	68 (74.7)	72 (71.3)	0.287	0.592
Course, years	6.0 (3.0, 10.0)	8.0 (3.0, 10.5)	−1.246	0.213
Chronic migraine	27 (29.7)	22(21.8)	1.567	0.211
Migraine with aura	22 (24.2)	22 (21.8)	0.155	0.694
Degree of RLS	3 (3, 4)	3 (3, 4)	−1.390	0.165
Constant RLS	60 (65.9)	73 (72.3)	0.905	0.341
Baseline HIT-6 scores	61.00 (58.00, 66.00)	62.00 (58.50, 66.00)	−0.972	0.331

*PFO, Patent foramen ovale*.

### Proportions of Chronic Migraine and Patients Under Preventive Treatments

At 1-year follow-up, 14 (15.4%) patients in the PFO closure group and 27 (26.7%) patients in the non-PFO closure group presented with chronic migraine; at 5-year follow-up, 8 (8.8%) patients in the PFO closure group and 20 (19.8%) patients in the non-PFO closure group presented with chronic migraine (Table 2). In terms of the preventive treatments, 39 (46.4%) patients in the PFO closure group and 49 (48.5%) patients in the non-PFO closure group used Chinese traditional medicine at 1-year follow-up; at 5-year follow-up, these figures were 24 (30.8%) in the PFO closure group and 36 (43.4%) in the non-PFO closure group. Only a few patients used β blockers or calcium channel blockers. None of the patients used anti-depressive or antiepileptic drugs. No significant difference has been observed between the two groups (Table 3).

**Table 2:** The proportion of chronic migraine and episodic migraine during the follow-up.

	**PFO closure group (*****n*** **=** **91)**		**non-PFO closure group (*****n*** **=** **101)**	
	**1-year follow-up**	**5-year follow up**	**1-year follow-up**	**5-year follow up**
Chronic migraine	14 (15.4)	8 (8.8)	27 (26.7)	20 (19.8)
Episodic migraine	49 (53.8)	31 (34.1)	68 (67.3)	37 (36.6)
Free of migraine	21 (23.1)	39 (42.9)	6 (6.0)	26 (25.7)
Lost to follow-up	7 (7.7)	13 (14.3)	0 (0.0)	18 (17.8)

*PFO, Patent foramen ovale*.

**Table 3:** Preventive medications used during the follow-up.

	**PFO closure group**	**non-PFO closure group**	**χ^2^**	***p***
**β BLOCKER**				
1-year follow-up	4 (4.8)	8 (7.9)	0.754	0.385
5-year follow-up	2 (2.6)	4 (4.8)	0.115	0.735
**CALCIUM CHANNEL BLOCKER**				
1-year follow-up	6 (7.1)	5 (5.0)	0.100	0.752
5-year follow-up	4 (5.1)	6 (7.2)	0.051	0.822
**CHINESE TRADITIONAL MEDICINE**				
1-year follow-up	39 (46.4)	49 (48.5)	0.080	0.777
5-year follow-up	24 (30.8)	36 (43.4)	2.733	0.098

*PFO, Patent foramen ovale*.

### Changes in the Shunt Degree and Status of RLS After Transcatheter PFO Closure

**Figure 1:**
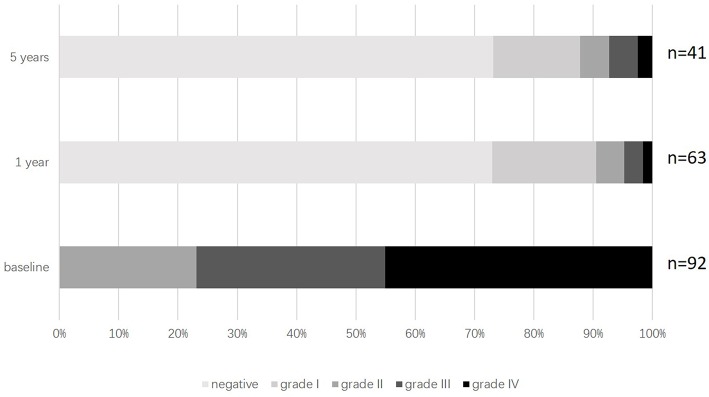
Distribution of RLS sizes during follow-up in PFO closure group. PFO, Patent foramen ovale; RLS, Right-to-left shunt.

Only patients in the PFO closure group were asked to reexamine c-TCD. Of 91 patients in the PFO closure group, 21 (22.8%) patients had grade II RLS, 29 (31.5%) patients had grade III RLS, and 41 (44.6%) patients had grade IV RLS before the PFO closure. Of 63 patients who returned for c-TCD examination at 1-year follow-up, 46 (73.0%) patients had no RLS, 11 (17.4%) patients had grade I RLS, three (4.8%) patients had grade II RLS, two (3.2%) patients had grade III RLS, and only one (1.6%) patient whose procedure was declared unsuccessful by the surgeon during the operation still had grade IV RLS. Of 41 patients who returned for c-TCD examination at 5-year follow-up, 30 (73.2%) patients had no RLS, sic (14.6%) patients had grade I RLS, two (4.9%) patients had grade II RLS, two (4.9%) patients had grade III RLS, and only one (2.4%) patient whose procedure was unsuccessful still had grade IV RLS (Figure 1).

### Changes in HIT-6 Scores of All Subjects

**Figure 2:**
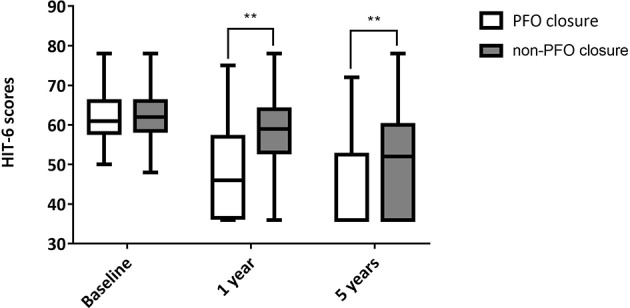
HIT-6 scores at different timepoints after baseline for all patients. **Indicates *p* < 0.001. PFO, Patent foramen ovale.

In the transcatheter PFO closure group, there were 84 (92.3%) and 78 (85.7%) patients who completed 1- and 5-year follow-up over telephone, respectively, while those numbers were 101 (100.0%) and 83 (82.2%) in the non-PFO closure group. The loss ratio at 5 years was 14.3% in the PFO closure group and 17.8% in the non-PFO closure group. There was a significant difference between HIT-6 scores in the two groups: 46.00 (36.50, 57.00) in PFO closure group vs. 59.00 (53.00, 64.00) in the non-PFO closure group at 1 year (*p* < 0.001), and 36.00 (36.00, 52.50) in the PFO closure group vs. 52.00 (36.00, 60.00) in the non-PFO closure group at 5 years (*p* < 0.001; Table 4, Figure 2).

**Table 4:** HIT-6 scores at different timepoints after baseline in all patients.

	**PFO closure group**	**non-PFO closure group**	**Z**	***p***
Baseline	61.00 (58.00, 66.00)	62.00 (58.50, 66.00)	−0.972	0.331
1 year	46.00 (36.50, 57.00)	59.00 (53.00, 64.00)	−5.888	<0.001
5 years	36.00 (36.00, 52.50)	52.00 (36.00, 60.00)	−3.628	<0.001

*PFO, Patent foramen ovale*.

### Changes in HIT-6 Scores of Subjects in Different Age Groups

**Figure 3:**
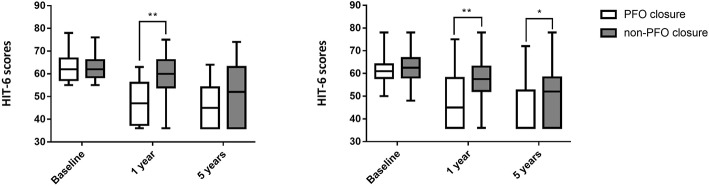
HIT-6 scores at different timepoints after baseline in different age groups. **(Left)** HIT-6 scores at different timepoints after baseline in patients older than 45 years; **(Right)** HIT-6 scores at different timepoints after baseline in patients younger than 45 years. **Indicates *p* < 0.001; *indicates *p* < 0.05. PFO, Patent foramen ovale.

In patients younger than 45 years, HIT-6 scores were statistically different between the two groups at both 1 and 5 years after baseline: 45.00 (36.00, 58.00) in the PFO closure group vs. 57.50 (52.25, 63.00) in the non-PFO closure group at 1 year (*p* < 0.001), and 36.00 (36.00, 52.50) in the PFO closure group vs. 52.00 (36.00, 58.25) in the non-PFO closure group at 5 years (*p* = 0.002). There was also a significant difference in HIT-6 scores of subjects older than 45 years at 1-year follow-up: 47.00 (37.50, 56.00) in the PFO closure group vs. 60.00 (54.00, 66.00) in the non-PFO closure group (*p* < 0.001). However, there was no significant difference in HIT-6 scores of subjects older than 45 years at 5-year follow-up: 45.00 (36.00, 54.00) in the PFO closure group vs. 52.00 (36.00, 63.00) in the non-PFO closure group (*p* = 0.067; Tables 5, 6, Figure 3).

**Table 5:** HIT-6 scores at different timepoints after baseline in patients whose age <45 years.

	**PFO closure group (*n* = 67)**	**non-PFO closure group (*n* = 64)**	**Z**	***p***
Baseline	61.00 (58.00, 64.00)	62.50 (58.25, 66.75)	−0.921	0.357
1 year	45.00 (36.00, 58.00)	57.50 (52.25, 63.00)	−4.251	<0.001
5 years	36.00 (36.00, 52.50)	52.00 (36.00, 58.25)	−3.137	0.002

*PFO, Patent foramen ovale*.

**Table 6:** HIT-6 scores at different timepoints after baseline in patients whose age ≥45 years.

	**PFO closure group (*n* = 24)**	**non-PFO closure group (*n* = 37)**	**Z**	***p***
Baseline	62.00 (52.75, 66.75)	62.00 (58.50, 66.00)	−0.244	0.807
1 year	47.00 (37.50, 56.00)	60.00 (54.00, 66.00)	−4.043	<0.001
5 years	45.00 (36.00, 54.00)	52.00 (36.00, 63.00)	−1.831	0.067

*PFO, Patent foramen ovale*.

### Changes in HIT-6 Scores of Subjects With Different Shunt Degree of RLS

**Figure 4:**
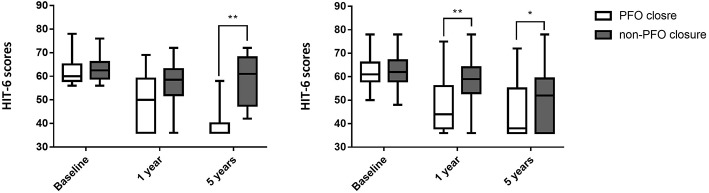
HIT-6 scores at different timepoints after baseline in patients with different degrees of RLS. **(Left)** HIT-6 scores at different timepoints after baseline in patients with moderate RLS; **(Right)** HIT-6 scores at different timepoints after baseline in patients with large RLS. **Indicates *p* < 0.001; *indicates *p* < 0.05. PFO, Patent foramen ovale; RLS, Right-to-left shunt.

In patients with moderate RLS, there was no significant difference in HIT-6 scores at 1-year follow-up between the two groups: 50.00 (36.00, 59.00) in the PFO closure group vs. 58.50 (52.00, 63.00) in the non-PFO closure groups (*p* = 0.068). At 5-year follow-up, however, statistically significant difference was observed between the two groups in patients with moderate RLS: 36.00 (36.00, 40.00) in the PFO closure group vs. 61.00 (47.50, 68.00) in the non-PFO closure group (*p* < 0.001). In patients with large RLS, HIT-6 scores were significantly different between the two groups at both 1- and 5-year follow-up: 44.00 (38.00, 56.00) in the PFO closure group vs. 59.00 (53.00, 64.00) in the non-PFO closure group at 1 year (*p* < 0.001), and 38.00 (36.00, 55.00) in the PFO closure group vs. 52.00 (36.00, 59.25) in the non-PFO closure group at 5 years (*p* = 0.019; Tables 7, 8, Figure 4).

**Table 7:** HIT-6 scores at different timepoints after baseline in patients with moderate RLS.

	**PFO closure group (*n* = 21)**	**non-PFO closure group (*n* = 10)**	**Z**	***p***
Baseline	60.00 (58.00, 65.00)	62.50 (59.00, 66.00)	−0.298	0.766
1 year	50.00 (36.00, 59.00)	58.50 (52.00, 63.00)	−1.828	0.068
5 years	36.00 (36.00, 40.00)	61.00 (47.50, 68.00)	−3.746	<0.001

*PFO, Patent foramen ovale; RLS, Right-to-left shunt*.

**Table 8:** HIT-6 scores at different timepoints after baseline in patients with large RLS.

	**PFO closure group (*n* = 70)**	**non-PFO closure group (*n* = 91)**	**Z**	***p***
Baseline	61.00 (58.00, 66.00)	62.00 (58.00, 67.00)	−0.950	0.342
1 year	44.00 (38.00, 56.00)	59.00 (53.00, 64.00)	−5.617	<0.001
5 years	38.00 (36.00, 55.00)	52.00 (36.00, 59.25)	−2.354	0.019

*PFO, Patent foramen ovale; RLS, Right-to-left shunt*.

## Discussion

According to 5-year follow-up, our results suggested that transcatheter PFO closure was effective in alleviating the disability of migraine. Moreover, we found an interesting effect of PFO closure on migraine in patients with different ages and different shunt degree of RLS. Subgroup analyses revealed that transcatheter PFO closure might not be as effective for 5-year follow-up in patients older than 45 years. Interestingly, although patients with moderate RLS did not benefit from this procedure at 1-year follow-up, transcatheter PFO closure did improve patients' symptoms at 5-year follow-up in this subgroup of patients.

Although the relationship between PFO and migraine remains controversial (24), increasing evidence in favor of this association has been reported. In particular, Wang et al. found that large RLS caused by PFO was strongly linked with migraine, while the proportion of mild and moderate RLS in migraineur did not differ from that in healthy individuals (9). This finding is consistent with our results: patients with large RLS benefitted from transcatheter PFO closure but patients with RLS in moderation did not at 1-year follow-up.

PFO, accounting for 95% of RLS (25), can be occluded through percutaneous/transcatheter PFO closure. The long-term efficacy of PFO closure in treating RLS was confirmed in our study as a dramatic decline in the size of RLS was observed and it did not revert to the previous level at the 5-year follow-up. However, prior randomized trials with respect to the efficacy of PFO closure in treating migraine have generated negative results. MIST trial randomized 147 subjects to transcatheter PFO closure group or to a sham surgery, and no significant difference was observed being reached between the two groups in terms of the migraine cessation during a 6-month follow-up. HIT-6 scores, as one of their secondary endpoints, were not different either over a 1-month retrospective period. However, the negative results of MIST might be due to the insufficient follow-up time because we found a significant HIT-6 scores difference between the two groups at both 1 and 5 years after the surgery. Patients in PREMIUM and PRIMA trials were required to record a headache diary to evaluate the severity of migraine, which could objectively reflect the change on the frequency of migraine and the duration of each migraine attack. However, given the current social and clinical circumstances in China, it is impossible to ask a patient to maintain a headache diary constantly for 5 years long. Alternatively, we employed HIT-6 questionnaire to assess the disability of migraine. Owing to the subjective nature of HIT-6 questionnaire, the HIT-6 scores are largely affected by the variability of pain tolerance in different individuals. Therefore, the increasing pain tolerance in older people may be responsible for the similar HIT-scores between the two groups in patients older than 45 years at 5-year follow-up in our study.

Considering the lack of accordant conclusions, an agreement has been reached by experts that PFO closure should not be recommended to prevent or treat migraine without strict selection of patients. Our results implied that the age of patients and the size of RLS need to be considered when selecting proper patients who might benefit from PFO closure as young migraineurs with a large size of RLS reported a better outcome after PFO closure in our study.

Our study has several limitations. First, as a retrospective study, we were unable to analyze more inter-group characteristics. This issue may be addressed in future prospective clinical trials. Second, the relatively small sample size in our study may make our data less stable. A multicenter study with more participants is needed in the future to generate more robust results.

To conclude, despite all aforementioned limitations, this study suggested that transcatheter PFO closure was effective in alleviating the disability of migraine with PFO, and this effect was more obvious when patients are younger than 45 years and the RLS are large in the long term.

## Data Availability Statement

The raw data supporting the conclusions of this manuscript will be made available by the authors, without undue reservation, to any qualified researcher.

## Ethics Statement

The studies involving human participants were reviewed and approved by The Ethics Committee of the First Hospital of Jilin University. The patients/participants provided their written informed consent to participate in this study.

## Author Contributions

YY and Z-NG contributed conception and design of the study. Y-DH, X-LY, and CQ collected and organized the database. Y-DH and PZ performed the statistical analysis. Y-DH wrote the first draft of the manuscript. Z-NG reviewed and revised the manuscript. All authors have read and approved the submitted version.

### Conflict of Interest

The authors declare that the research was conducted in the absence of any commercial or financial relationships that could be construed as a potential conflict of interest.
